# High sugar intake from sugar‐sweetened beverages is associated with prevalence of untreated decay in US adults: NHANES 2013–2016

**DOI:** 10.1111/cdoe.12725

**Published:** 2021-12-23

**Authors:** Mark E. Moss, Huabin Luo, Asher Y. Rosinger, Molly M. Jacobs, Roopwant Kaur

**Affiliations:** ^1^ ECU School of Dental Medicine East Carolina University Greenville North Carolina USA; ^2^ Brody School of Medicine East Carolina University Greenville North Carolina USA; ^3^ Department of Biobehavioral Health Pennsylvania State University State College Pennsylvania USA; ^4^ Department of Anthropology Pennsylvania State University State College Pennsylvania USA; ^5^ College of Public Health and Health Professions University of Florida Gainesville Florida USA

**Keywords:** dental caries, health policy, nutrition surveys, sugar‐sweetened beverages

## Abstract

**Objectives:**

To assess the association between sugar from sugar‐sweetened beverages (SSBs) and untreated decay in permanent teeth and calculate the cost burden of sugar from SSBs on untreated decay in US adults.

**Methods:**

Cross‐sectional data from the 2013–2014 and 2015–2016 cycles of the National Health and Nutrition Examination Survey (NHANES) were analysed in 2020 (*n* = 9001 adults aged ≥20). Multivariable analyses assessed sugar intake from SSB consumption with the presence of untreated decay in permanent teeth and number of untreated decayed teeth. Population attributable risk was used to estimate the cost burden arising from SSBs on untreated decay in US adults.

**Results:**

One fourth (25.1%) of US adults had untreated dental decay, and higher prevalence was observed among those with low income, low education and race/ethnicity of non‐Hispanic Black. Overall, 53% of adults reported no intake of SSBs. For the remaining 47%, the median 24‐h intake was 46.8 g of sugar from SSBs. The adjusted prevalence ratio (PR) for untreated decay was 1.3 (95% confidence interval [CI] 1.1–1.5) for consumption of 46.8 g or more of sugar from SSBs compared to those reporting no sugar from SSBs. Number of untreated decayed teeth increased with sugar intake from SSBs from lowest to highest tertile: 0.1, (*p *= .35); 0.4, (*p *= .006); and 0.6, (*p *< .001). The cost burden of untreated decay attributable to SSBs in US adults is estimated conservatively at $1.6 billion USD.

**Conclusions:**

Community level interventions directed at sugar from SSBs are justified to address disparities in the burden of untreated dental decay.

## 1 INTRODUCTION

Untreated dental decay is recognized as a global problem affecting all age groups.^1^, ^2^ This condition represents not only the outcome of a multifactorial disease process (dental caries), the lack of treatment can also indicate a failure to meet the need for dental care. In many respects, the burden of untreated dental decay in a population can frame a call to action for all stakeholders in the dental service delivery system.^3^ In order to make appropriate policy changes, population‐level assessments that account for social and demographic factors must be examined with respect to untreated dental decay.^4^, ^5^, ^6^, ^7^ This paper provides actionable data to drive policy development around the role of grams of sugar from sugar‐sweetened beverages (SSBs) and untreated dental decay using population‐level data for adults in the United States.

Published research has made a strong case for a causal association between sugar from SSBs and dental caries.^8^ As a disease process, dental caries is the interplay of diet and bacterial factors to create a low pH microenvironment where demineralization of tooth structure outpaces remineralization over time.^9^ Undoubtedly, fermentable carbohydrates provide a causal pathway for dental caries.^10^, ^11^, ^12^ Studies have established the importance of sugar intake from beverages.^8^, ^13^ While sugar from other sources (i.e., candy, bakery items and chewing gum) and other fermentable carbohydrates contribute to dental decay, sugar‐sweetened beverages (SSBs) have been identified by policy makers as a focus for action.^14^, ^15^, ^16^, ^17^, ^18^, ^19^, ^20^ It should be noted that in addition to the role sugar plays in dental disease, the World Health Organization has recommended reductions in sugar intake for adults and children to address a broad range of health issues arising from a role for sugar in poor dietary quality and obesity.^21^

In 2019, expenditures for dental care in the United States were estimated at $143 billion (US dollars).^22^ Unmet need for treatment represents an additional burden that is not captured in expenditure data. Therefore, this study investigated the association between sugar from SSBs and untreated dental decay using data from the 2013–2016 National Health and Nutrition Examination Survey (NHANES). The aim of this study was twofold. First, to test the association between grams of sugar consumed from SSBs and (a) the presence or absence of untreated decay; and (b) the number of untreated decayed teeth. Second, in order to frame the policy implications, population attributable risk (PAR) per cent was used to estimate treatment costs. As a policy tool,^23^ PAR provides a simple estimation of the burden of untreated dental decay attributable to sugar from SSB consumption among US adults.

## 2 METHODS

**FIGURE 1:**
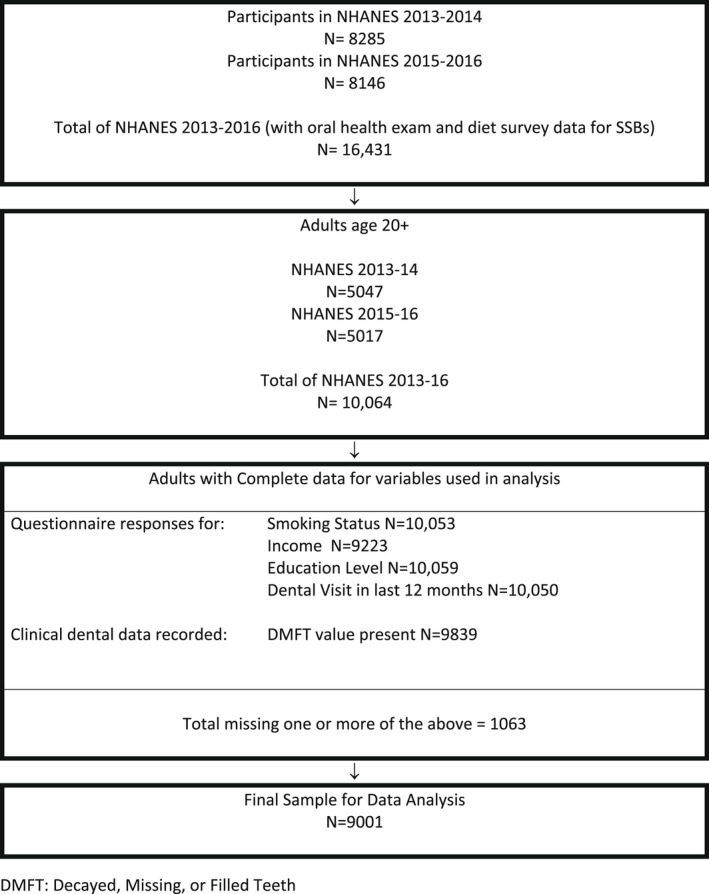
Flow diagram for study inclusion

Figure 1 shows how the study sample was derived in 2019 from the public‐use data files for two cycles of NHANES: 2013–2016. NHANES is a public‐use de‐identified data set containing information on behavioural, demographic and environmental characteristics. In additional to these characteristics, the cross‐sectional NHANES survey conducted household interviews and standard oral health examinations. All observations were weighted to a representative sample of the non‐institutionalized, civilian, household population of the United States.

### 2.1 Procedures

#### 2.1.1 Outcomes

Analysis was limited to persons 20 years and older with at least 1 permanent tooth. For these individuals, each tooth surface was examined and scored in NHANES by calibrated, licensed dentists.^24^ Tooth level scores were summed to provide an individual score representing the number of permanent decayed, missing and filled teeth (DMFT). In the dental protocol, untreated decay was assigned when cavitation at the dentine level was present at the examination. In this study, permanent tooth decay status was summarized as (a) presence or absence of untreated decay; and (b) number of untreated decayed teeth.

#### 2.1.2 Primary predictor: sugar from SSBs

NHANES obtained 24‐h multiple pass dietary recall data from in‐person interviews. Dietary data files were sent electronically from the field and were imported into Survey Net, a computer‐assisted food coding and data management system developed by United States Department of Agriculture (USDA).^25^

An algorithm to convert the reported beverages and their sugar content was used to link to USDA's Food and Nutrient Database for Dietary Studies (FNDDS). The basis for the nutrient values in FNDDS is detailed in the documentation and available at http://www.ars.usda.gov/nea/bhnrc/fsrg.

Following previous analyses,^26^ SSBs included regular soda, sports and energy drinks, sweetened coffees and teas, fruit drinks (including sweetened bottled waters and fruit juices), and other beverages fitting this definition (including horchata and sugar cane beverages). SSBs did not include diet drinks, which were defined as approximately, <40 kcal/240 ml of the beverage; 100% fruit juices; beverages sweetened by the participant, including coffee and teas; alcohol; or flavoured milks. For beverages that met the criteria of SSBs, the total number of grams of sugar from the SSBs per participant was summed over the 24‐h dietary recall period.

Grams (g) of sugar from SSBs was assessed as a categorical variable in two ways, using four levels to examine the presence of a dose‐response pattern and using three levels to simplify the findings for policy development. In both approaches, intake of 0 g of sugar from SSBs was used as the reference group and values were rounded to one decimal place for simplicity of presentation. At four levels, the study sample with >0 intake of sugar from SSBs was defined by using tertiles. The four levels were zero, >0 but <34.8 g, 34.8 g to 73.9 g, and 74.0 g or greater. The data pattern was simplified to three levels for the calculation of population attributable risk, by splitting the group with non‐zero intake of sugar from SSBs at the median. The three levels for this were as follows: 0 g of sugar from SSBs; more than 0 but <46.8 g; and 46.8 g or more of sugar from SSBs. As a frame of reference, a typical 12 oz sugar‐sweetened soft drink contains 39 g of sugar and a 20 oz soft drink contains 65 g of sugar.

Covariates of interest available in NHANES were smoking status, age, self‐reported race/ethnicity, gender, household income level as measured through the federal poverty income ratio—a ratio of family income to poverty guidelines—as ≤130%, 131%–350% and >350%, education level (identified by highest level attained: less than high school, high school graduation, some college, college and above), and dental visit behaviour (as whether or not there was a dental office visit in the past 12 months). Smoking status was self‐reported as current, former or never.

### 2.2 Statistical analysis

Methods to account for the sampling weights were incorporated into data analysis by using STATA version 13.1. Untreated decay was captured in two ways: (a) a binary value indicating the presence of any decay and (b) a count indicating the total number of untreated decayed teeth.

The association between sugar from SSBs and the presence of any untreated decay was tested by using a generalized linear model with a Poisson distribution and log‐link function regression with a binary dependent variable. Prevalence ratios were calculated by exponentiating regression coefficients. Negative binomial regression was used to test the relationship between sugar from SSBs and the number of teeth with untreated decay. To illustrate the predicted values of these two outcomes by sugar consumption level, graphs were created to show post‐estimation marginal standardization for regressions adjusting for the distribution of other covariates.^27^ All analyses used the Day 1 dietary sampling weights to account for the complex survey design of NHANES as well as oversampling, non‐response, noncoverage and day of the week.

The PAR per cent serves as a policy tool that provides an estimate of the amount of untreated decay that could be averted if sugar intake from SSBs was reduced.^23^ To be meaningful the PAR assumes that a causal relationship has been established between the risk factor and disease outcome. Given this assumption, PAR requires valid estimates of exposure in the population of interest and estimate of relative risk. A simple estimate of the economic cost for treatment of untreated dental decay was computed by using values from the 2013 Survey of Dental Fees conducted by the American Dental Association (https://www.aapd.org/assets/1/7/PolicyCenter‐2013_Survey_of_Dental_Fees.pdf) based on a periapical radiograph, and a simple one‐surface amalgam restoration. Population estimates were obtained from US Census estimates for adults aged 20 and older available on the Census Reporter website (https://censusreporter.org/profiles/01000us‐united‐states/). The average for 2013–2016 was used to compute number of adults with untreated decay in the US population. The adjusted prevalence rate ratio obtained from regression results was used to derive an estimate of relative risk (*RR*) using consumption of 46.8 g of sugar from SSBs as this represented the median among US adults who reported consuming SSBs. Population attributable risk per cent^28^ estimated the number of adults in the United States with untreated decay that is attributable to sugar intake from SSBs. The formula uses estimates of *RR* and prevalence of exposure (*p*) as follows:

PopulationAttributableRiskPercent=(p∗(RR‐1)/((p∗(RR‐1)+1)

The relative increment in number of untreated decayed teeth was derived from the negative binomial model.^29^ In this manner, a conservative estimate of treatment costs to address a single surface lesion attributable to sugar from SSBs was derived for US adults where the base cost would be $322 to restore one tooth and an additional $125 to restore a second tooth at the same visit. These estimates include the fee for a comprehensive examination and full‐mouth series of radiographs.

## 3 RESULTS

The sample consisted of 9001 adults with complete data for the variables of interest (Figure 1). Table 1 shows the distribution of the study sample. Over 50 per cent of adults consumed no sugar from SSBs (53.2%, 95% confidence interval (CI): 51.0, 55.4). About 25 per cent of the US adult population had at least one tooth with untreated caries. Only about 8 per cent (95% CI: 7.4, 8.9) of the US adult population had never had any dental decay. The mean number of untreated decayed teeth was 0.8 (95% CI:0.7, 0.9). On average in the United States, adults had about 11 decayed, missing and filled (DMF) permanent teeth (95% CI:10.8, 11.4).

**TABLE 1:** Selected characteristics of the study participants

Characteristic	*n* ^a^	%, Mean (weighted)	95% CI	
Age (years)				
20–44	3918	44.8	42.2	47.4
45–64	3064	35.9	33.9	38.1
65+	2019	19.2	17.7	20.8
Sex				
Female	4651	51.4	50.2	52.6
Male	4350	48.6	47.4	49.8
Race/ethnicity				
Non‐Hispanic white	3660	66.6	61.5	71.3
Non‐Hispanic black	1836	10.8	8.4	13.7
Non‐Hispanic Asian	914	5.4	4.1	7.1
Hispanic	2291	14.0	10.9	17.8
Other	300	3.2	2.6	4.0
Federal poverty income ratio^b^				
≤130%	2935	22.8	19.9	26.1
131%–350%	3372	35.7	33.7	37.8
>350%	2694	41.4	37.5	45.5
Education level				
Less than high school	1845	13.5	11.4	15.9
High school	2018	21.6	19.8	23.6
Some college	2801	33.4	31.6	35.3
College and above	2337	31.5	27.9	35.3
Dental visit in past 12 months				
Yes	4949	60.0	57.0	62.9
No	4052	40.0	37.1	43.0
Smoking status				
Current	1763	18.7	17.3	20.2
Former	2160	25.3	23.7	26.9
Never	5078	56.0	54.1	57.8
Sugar from SSB consumption				
None (0 g of sugar)	4465	53.2	51.0	55.4
More than 0 but <38.4 g	1560	15.2	14.1	16.5
38.4 g to <74.0 g	1606	16.9	15.8	18.1
74.0 g or more	1370	14.6	13.1	16.3
Untreated dental caries present				
Yes	2617	25.1	22.9	27.5
No	6384	74.9	72.5	77.1
Any Decayed, Missing, or Filled (DMF) teeth				
Yes	8281	91.9	91.1	92.6
No	720	8.1	7.4	8.9
# of teeth with untreated caries (mean)	9001	0.8	0.7	0.9
# DMF teeth (mean)	9001	11.1	10.8	11.4

National Health and Nutrition Examination Survey (NHANES) 2013–2016. *n* = 9001.

^a^ Unweighted sample size.

^b^ Federal poverty income ratio is derived by scaling the household income relative to the level defined as the US Federal Poverty Level for that year based on number of persons in the household.

Regression results are displayed in Table 2. The first set of columns present prevalence ratios (PRs) and 95 per cent CIs for the Poisson likelihood of having at least one tooth with untreated decay. The last set of columns present the regression results for a negative binomial model estimating the association with mean number of untreated decayed teeth. Both models show consistent results.

**TABLE 2:** Multivariable models for association between sugar intake from sugar‐sweetened beverages and selected confounders with untreated decay among US Adults, NHANES 2013–2016

*N* = 9001	Presence of untreated dental caries (yes/no)				Number of untreated decayed teeth			
Characteristic	Adjusted Prevalence Ratio	95% Confidence Interval Upper limit Lower limit		*p *> *t*	Coefficient	95% Confidence Interval Upper limit Lower limit		*p *> *t*
Sugar from SSB consumption (vs. None)								
More than 0 and <34.8 g	1.1	0.90	1.3	.44	0.1	−0.1	0.4	.35
34.8 g to <74.0 g	1.1	0.99	1.3	.07	0.4	0.1	0.6	.006
74.0 g or more	1.4	1.2	1.6	<.001	0.6	0.4	0.8	<.001
Smoking (vs. Never Smoking)								
Former	1.1	0.95	1.2	.27	0.1	−0.1	0.3	.34
Current	1.3	1.2	1.5	<.001	0.6	0.4	0.8	<.001
Age (vs. 20–44)								
45–64	0.95	0.84	1.1	.39	−0.2	−0.4	0.0	.11
65+	0.70	0.57	0.85	.001	−0.5	−0.8	−0.2	.002
Female	0.90	0.80	1.0	.09	−0.3	−0.5	−0.1	.01
Race/ethnicity (vs. non‐Hispanic white)								
Non‐Hispanic black	1.4	1.2	1.6	<.001	0.4	0.2	0.5	<.001
Non‐Hispanic Asian	0.96	0.77	1.2	.74	0.1	−0.3	0.5	.69
Hispanic	1.1	0.97	1.3	.13	0.1	−0.2	0.3	.66
Other	1.1	0.79	1.4	.71	0.1	−0.3	0.5	.60
Poverty income ratio (vs. ≤130%)								
131%–350%	0.89	0.80	1.0	.05	−0.2	−0.3	−0.1	.001
>350%	0.59	0.52	0.68	<.001	−0.9	−1.1	−0.7	<.001
Education (vs. less than high school)								
High school	0.97	0.87	1.1	.41	0.0	−0.2	0.2	.93
Some college	0.80	0.70	0.91	.002	−0.3	−0.5	−0.1	.002
College and above	0.54	0.44	0.67	<.001	−0.8	−1.1	−0.6	<.001
Dental visit in past year (vs. no)	0.57	0.51	0.65	<.001	−0.7	−0.9	−0.6	<.001

^a^ Poisson regression model (first set of columns);

^b^ Negative binomial regression model (last four columns).

**FIGURE 2:**
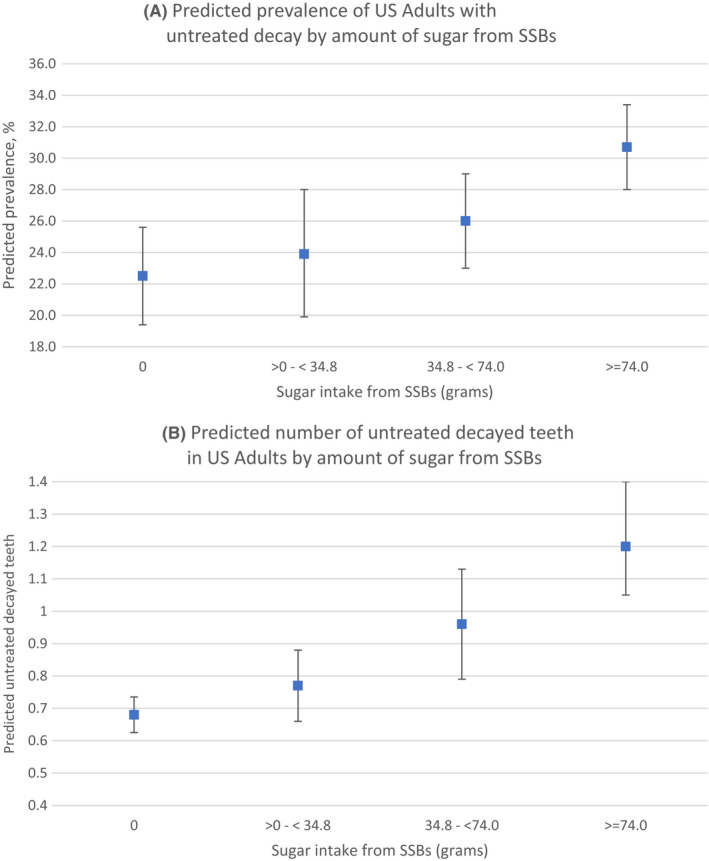
Predicted values for Prevalence of (A) Untreated Decay and (B) Number of Teeth with Untreated Decay in US Adults by amount of sugar intake from sugar‐sweetened beverages (SSBs): NHANES 2013–2016 (*n* = 9001)

Those consuming high levels of sugar intake from SSBs had a higher prevalence of untreated decay compared to those reporting no sugar intake from SSBs. The adjusted prevalence ratio was 1.4 (95% CI: 1.2, 1.6) for those consuming 74.0 g or more compared to those consuming no sugar from SSBs. The number of untreated decayed teeth was positively associated with the amount of sugar intake from SSBs: 0.4 (95% CI: 0.1, 0.6) at intake of 34.8 to 74.0 g; and 0.6 (95% CI: 0.4, 0.8) at 74.0 g or more relative to an expected mean in those who consume no sugar from SSBs. Figure 2 shows predicted values for the outcomes with increased sugar from SSBs on prevalence untreated decay (Figure 2 panel A) and number of untreated decayed teeth (Figure 2 panel B).

The models (Table 2 **)** show that adults aged 65 and older were less likely to have untreated decayed teeth than adults aged 20–44 years (PR 0.70, 95% CI: 0.57, 0.85). They also had statistically lower numbers of untreated decayed teeth (*p *= .002). Moreover, non‐Hispanic Black adults had a higher likelihood of untreated decay relative to non‐Hispanic whites after controlling for income and education (PR 1.4, 95% CI: 1.2, 1.6). Socioeconomic status showed strong ‘dose‐response’ associations with untreated decay—those with higher income relative to poverty (high income PR 0.59, 95% CI: 0.52, 0.68 and moderate income PR 0.89, 95% CI: 0.80, 1.0 compared to low income) and more education (College and above PR 0.54, 95% CI: 0.44, 0.67 and some college PR 0.80, 95% CI: 0.70, 0.91 compared to less than high school education) had lower prevalence of untreated decay. Individuals who reported having a dental visit in the past year were less likely to have untreated decay (PR 0.57, 95% CI: 0.51, 0.65). Current smokers were more likely to have untreated decay relative those who reported never smoking (PR 1.3, 95% CI: 1.2, 1.5).

PAR results shown in Table 3 were estimated by using the prevalence ratio from a multivariable model with non‐zero sugar intake divided at the median. In this model, the adjusted prevalence was 30 per cent greater for untreated decay for those consuming more than the median amount of sugar from SSBs compared to those consuming no sugar from beverages (PR 1.3, 95% CI: 1.1, 1.5). PAR results suggest that 6.8 per cent of US adults had untreated decay attributable to sugar intake from SSBs (Table 3). This translates to 3.6 million people who had untreated decay because of high SSB consumption. Estimates from the negative binomial model show that high SSB consumption contributed to an average of two additional decayed teeth among those with untreated decay. Table 3 shows that the cost of treatment can be estimated from these data. The economic impact is conservatively estimated at $1.6 billion (USD).

**TABLE 3:** Population Attributable Risk (PAR) per cent for Sugar from SSBs and untreated dental decay among US Adults

Estimated RR at exposure level	Prevalence of exposure to high sugar (46.8 g or more) from SSB intake	% of cases of untreated decay in the US Adult Population attributable to SSBs	Estimated number of adults with untreated decay attributable to SSBs	Estimated number of teeth with untreated decay attributable to SSBs	Estimated costs to restore these teeth
1.3	24.2%	6.8%	3 660 328	7 320 656	$1.6 billion

PAR Calculation Details						
Sugar from SSB consumption (vs. None)	Adjusted^a^ Prevalence Ratio	95% Confidence Interval	Adjusted^a^ Risk Increase in Expected Mean among those with non‐zero untreated decayed teeth	95% Confidence Interval		
Moderate (<46.8 g)	1.1	0.91–1.2	1.2	0.9–1.4		
High (46.8 g or more)	1.3	1.1–1.5	1.7	1.4–2.1		

Economic Cost Estimates:	Examination (D0150)	Radiographs (D0220)	Single Restoration (D2140)	Base Dental Services (Charges)	1 additional restoration at same visit
Cost in terms of dental fees	$73	$124	$125	$322	$125

Note

NHANES 2013–2016. *N* = 9001. Dental fees vary for specific procedures. Average fee data were used from the 2013 ADA survey to estimate costs https://www.aapd.org/assets/1/7/PolicyCenter‐2013_Survey_of_Dental_Fees.pdf.

^a^ Adjusted for same variables as listed in Table 2 (smoking, age, sex, race/ethnicity, poverty income ratio, education and dental visit in past year).

## 4 DISCUSSION

These analyses support the hypotheses that grams of sugar from SSBs are positively associated with the presence of any untreated decayed teeth and those who consume more grams of sugar from SSBs have more teeth with untreated decay. This is especially evident in the negative binomial model where those consuming 34.8 to 74.0 g and those consuming more than 74.0 g had coefficients of 0.4 (95% CI: 0.1, 0.6) and 0.6 (95% CI: 0.4, 0.8) respectively. The PAR model attributes 6.8 per cent of cases of untreated decay in US adults to consumption of more than the median amount of sugar from SSBs. A simple estimate shows that this translates to about $1.6 billion (USD) in treatment costs. To place this into context, the 2019 budget for the Division of Oral Health at the US Centers for Disease Control and Prevention was $19.5 million (USD).^30^

These cross‐sectional findings are consistent with prospective findings for sugar from SSBs^13^ as well as social determinants^31^, ^32^, ^33^ and smoking.^34^, ^35^ Our results show that untreated decay follows strong socioeconomic indicators—those with low income, and low education have higher burdens. Also, those who are non‐Hispanic Black have burdens that are significantly greater than those who are non‐Hispanic White. These results are consistent with prior work that shows that tap water avoidance, which leads to SSB consumption is highest in these groups due to marginalization and histories of inequitable access to clean water.^36^ While further validation of these findings in longitudinal studies is needed, sufficient evidence exists to justify taxes based on grams of sugar in SSBs and to monitor the results. Recommendations associated with added sugars are usually expressed in kilocalories (kcals). For example, the American Heart Association (AHA) recommends limiting added sugar consumption to 100 kcals (equivalent to 25 g) for women and 150 kcals (~36 g) for men,^37^ while the USDA recommends limiting the percentage of total calories from added sugars at 10%.^38^ Our findings support limiting added sugar consumption from SSBs below 35 g. Our use of an estimate of risk based on grams of sugar makes a strong case for a focus on sugar itself and not simply the behaviours associated with consumption of SSBs; however, some residual confounding cannot be ruled out.

Dental caries is a multifactorial disease process and while the literature clearly links sugar exposure to dental caries, only a few observational studies provide evidence among adults.^8^ Work in Finland has established that sugar intake is linked to adult caries increment in a linear dose‐response manner.^39^ Two recent studies used data from NHANES. One focused on US adults in the 2013–2014 cycle of NHANES and showed an association in those older than age 30 between dental caries experience measured as DMFT and dietary patterns that favoured SSBs and sandwiches.^40^ The second study^41^ examined SSB consumers relative to those reporting no consumption of SSBs across various age groups for data collected in 2011–2014. They found that SSB users had a higher prevalence of untreated decay. Our study includes adults age 20 and older from both the 2013–2014 and 2015–2016 cycles of NHANES. Our analysis aligns with policies proposing a tax based on grams of sugar in SSBs.^16^

The concept of a tax on sugar from SSBs, based largely on beneficial effects arising from reduced intake of sugar on the population, has been widely promoted in public health policy circles.^14^, ^15^, ^16^, ^20^ However, critics argue that such a tax has the potential to be ‘regressive’ by adversely impacting the segment of the population that is least able to bear the additional cost. Allcott et al.^14^ point out that policy can be tailored so that revenues from the tax are ear‐marked for programmes that can benefit low‐income populations, as was done in Philadelphia.^17^

This study used data from a large, nationally representative sample of the US adult population with standardized measures for dental caries and grams of sugar from SSBs. This provides an opportunity for estimating population‐level burden using accurate estimates of prevalence and exposure. Our findings for untreated dental decay support the recommendations from both the AHA and USDA to limit sugar consumption to below 35 g of sugar. Furthermore, we found a dose‐response association for a higher prevalence of untreated decay in US adults and a higher predicted number of untreated decayed teeth for those with more than 35 g of sugar from SSBs. Limitations in this study include use of cross‐sectional data and self‐reported 24‐h dietary recall. Total sugar and frequency of consumption are highly correlated, and this study cannot disaggregate these factors. We cannot rule out that frequency of consumption is more important than total amount of sugar consumed.^12^ Also, frequency of SSB consumption has been linked with permanent tooth loss among U.S. young adults,^42^ and our analysis does not account for missing teeth attributable to SSB intake. Likewise, sugar from other sources and healthy behaviours such as toothbrushing and fluoride exposure are not accounted for in our study. Our economic cost estimation does not attempt to capture any indirect costs attributable to untreated decay or model the implications over time as has been done by others.^18^, ^19^, ^43^, ^44^ We used an estimate for costs that was based solely on average fees for selected services from 2013 and this came to $477 per person affected. These costs may not be realistic; however, it is relevant that Manski and Rohde estimated the average expense per person in the United States for dental services in 2015 was $486.14 for those with only public insurance.^45^ Future research should examine economic costs and patterns of care in more detail.

Untreated decay is used widely for international comparisons.^1^, ^2^ However, presence of untreated decayed teeth represents a constellation of at least three components—a disease process; a behavioural component; and the ability to obtain dental care when sought. Dental care seeking was measured by dental visits in the past year. While significant, we are only able to speculate on the intersectionality of socioeconomic status, care‐seeking behaviour, and access to care.^46^ This study is unable to completely untangle these components.

## 5 CONCLUSION

The burden of untreated decay attributable to intake of sugar from SSBs is clearly present in the US adult population identified as having a low income, low education attainment and/or who identified as non‐Hispanic Black. Treatment costs attributable to SSB consumption exceed $1.6 billion in US adults. As a policy lever, revenue generated by a tax on SSBs can be justified to support local communities in addressing the social inequities that contribute to untreated dental decay in underserved population groups.

## CONFLICT OF INTEREST

None to declare.

## AUTHOR CONTRIBUTIONS

MEM, HL and AYR contributed to the conception, design, data acquisition and initially drafted the approach for this paper. HL conducted the data analysis and provided interpretation of the findings. AYR provided specific guidance and interpretation for the computation of sugar from beverages. MMJ provided the context and interpretation of aspects specific to health economics. RK provided the interpretation of aspects specific to clinical caries. In this manner, all authors (MEM, HL, AYR, MMJ and RK) contributed to the scientific content and to the critical revision of the manuscript including the figures and tables. All authors gave their final approval and agree to be accountable for all aspects of the work.

## Data Availability

NHANES is publicly available. Requests for access to the methods for computing grams of sugar from SSB intake can be directed to AYR.
